# Hyperuricemia as a Marker of Reduced Left Ventricular Ejection Fraction in Patients with Atrial Fibrillation: Results of the POL-AF Registry Study

**DOI:** 10.3390/jcm10091829

**Published:** 2021-04-22

**Authors:** Marcin Wełnicki, Iwona Gorczyca, Wiktor Wójcik, Olga Jelonek, Małgorzata Maciorowska, Beata Uziębło-Życzkowska, Maciej Wójcik, Robert Błaszczyk, Renata Rajtar-Salwa, Tomasz Tokarek, Jacek Bil, Michał Wojewódzki, Anna Szpotowicz, Małgorzata Krzciuk, Monika Gawałko, Agnieszka Kapłon-Cieślicka, Anna Tomaszuk-Kazberuk, Anna Szyszkowska, Janusz Bednarski, Elwira Bakuła-Ostalska, Beata Wożakowska-Kapłon, Artur Mamcarz

**Affiliations:** 1 3rd Department of Internal Medicine and Cardiology, Medical University of Warsaw, 02-091 Warsaw, Poland; welnicki.marcin@gmail.com (M.W.); wpwojcik@gmail.com (W.W.); a.mamcarz@3med.pl (A.M.); 2 1st Clinic of Cardiology and Electrotherapy, Swietokrzyskie Cardiology Centre, 25-736 Kielce, Poland; olga_jelonek@wp.pl (O.J.); bw.kaplon@poczta.onet.pl (B.W.-K.); 3 Collegium Medicum, The Jan Kochanowski University, 25-369 Kielce, Poland; 4 Department of Cardiology and Internal Diseases, Military Institute of Medicine, 04-141 Warsaw, Poland; mmaciorowska@wim.mil.pl (M.M.); buzieblo-zyczkowska@wim.mil.pl (B.U.-Ż.); 5 Department of Cardiology, Medical University of Lublin, 20-059 Lublin, Poland; m.wojcik@umlub.pl (M.W.); robertblaszczyk1@wp.pl (R.B.); 6 Department of Cardiology and Cardiovascular Interventions, University Hospital, 30-688 Krakow, Poland; rajfura@op.pl (R.R.-S.); tomek.tokarek@gmail.com (T.T.); 7 Department of Invasive Cardiology, Centre of Postgraduate Medical Education, Central Clinical Hospital of the Ministry of Interior and Administration, 02-507 Warsaw, Poland; biljacek@gmail.com (J.B.); michaljerzywojewodzki@gmail.com (M.W.); 8 Department of Cardiology, Regional Hospital, 27-400 Ostrowiec Swiętokrzyski, Poland; szpotowiczanna@wp.pl (A.S.); krzciukm@gazeta.pl (M.K.); 9 1st Chair and Department of Cardiology, Medical University of Warsaw, 02-097 Warsaw, Poland; mgawalko@wum.edu.pl (M.G.); agnieszka.kaplon@gmail.com (A.K.-C.); 10 Department of Cardiology, Medical University, 15-276 Bialystok, Poland; a.tomaszuk@poczta.fm (A.T.-K.); annaszyszkowska92@gmail.com (A.S.); 11 Department of Cardiology, St John Paul II Western Hospital, 05-825 Grodzisk Mazowiecki, Poland; medbed@wp.pl (J.B.); elwira.bakula@gmail.com (E.B.-O.)

**Keywords:** hyperuricemia, atrial fibrillation, heart failure, left ventricular ejection fraction

## Abstract

**Background**: Hyperuricemia is an established risk factor for cardiovascular disease, including atrial fibrillation (AF). The prevalence of hyperuricemia and its clinical significance in patients with already diagnosed AF remain unexplored. **Methods**: The Polish Atrial Fibrillation (POL-AF) registry includes consecutive patients with AF hospitalized in 10 Polish cardiology centers from January to December 2019. This analysis included patients in whom serum uric acid (SUA) was measured. **Results**: From 3999 POL-AF patients, 1613 were included in the analysis. The mean age of the subjects was 72 ± 11.6 years, and the mean SUA was 6.88 ± 1.93 mg/dL. Hyperuricemia was found in 43% of respondents. Eighty-four percent of the respondents were assigned to the high cardiovascular risk group, and 45% of these had SUA >7 mg/dL. Comparison of the extreme SUA groups (<5 mg/dL vs. >7 mg/dL) showed significant differences in renal parameters, total cholesterol concentration, and left ventricular ejection fraction (EF). Multivariate regression analysis showed that SUA >7 mg/dL (OR 1.74, 95% CI 1.32–2.30) and GFR <60 mL/min/1.73 m^2^ (OR 1.94, 95% CI 1.46–2.48) are significant markers of EF <40% in the study population. Female sex was a protective factor (OR 0.74, 95% CI 0.56–0.97). The cut-off point for SUA with 60% sensitivity and specificity indicative of an EF <40% was 6.9 mg/dL. **Conclusions**: Although rarely assessed, hyperuricemia appears to be common in patients with AF. High SUA levels may be a significant biomarker of reduced left ventricular EF in AF patients.

## 1. Introduction

In recent years, hyperuricemia has become a subject of interest to cardiologists and internists [1,2,3,4]. After many years in which high serum uric acid (SUA) levels in gout-free patients were ignored and only SUA levels above 12 mg/dL were considered significant, it has been noticed that significantly lower SUA levels may be associated with an increase in cardiovascular risk. [1,2,3,4]. Currently, the upper limit of normal for SUA levels is 7 mg/dL (420 µmol/L) for men and 6 mg/dL (360 µmol/L) for women. At the same time, in patients at high cardiovascular risk, the suggested goal is concentrations below 5 mg/dL (297 µmol/L) [4,5]. The cause of hyperuricemia is excessive endogenous uric acid production and decreased uric acid (UA) excretion [4,5]. Overproduction associated with excessive xanthine oxidase (XO) activity may be a consequence of a diet rich in purines and fructose, but also of metabolic disorders and genetic conditions [4,5]. The impaired excretion of UA is, in turn, mainly related to kidney function, but may also be the result of the use of certain drugs (e.g., diuretics, non-steroidal anti-inflammatory drugs, etc.) [4,5]. In the context of risk factors and diseases of the cardiovascular system, the relationship between hyperuricemia and the occurrence of dyslipidemia, carbohydrate metabolism disorders, abdominal obesity, and metabolic syndrome has so far been confirmed [6,7]. The PAMELA study proved that an increase in SUA concentration by 1 mg/dL increases the risk of arterial hypertension by approximately 30% [8]. In a meta-analysis of 18 studies on hyperuricemia and hypertension, it was proved that a 1% increase in SUA concentration translates into a 13% increase in the risk of hypertension [9]. It has also been proven that hyperuricemia increases the risk of stroke, including fatal stroke [10,11]. Finally, the negative prognostic significance of hyperuricemia in patients with acute coronary syndrome has been confirmed. Ndrepepa et al., assessing the prognosis of more than 5000 patients after acute coronary syndromes, showed that every 1 mg/dL increase in the SUA concentration increases the annual risk of death by 12% [12]. Ilundain-González et al. demonstrated that in patients with type 2 diabetes, each 1 mg/dL increase in SUA concentration is associated with a 30% increase in the risk of death [13]. Many studies have also shown that hyperuricemia increases the risk of developing atrial fibrillation [14,15]. However, according to the best knowledge of the authors of the POL-AF registry, the significance of increased SUA concentration in patients with already diagnosed atrial fibrillation has yet to be studied. Our analysis was aimed at determining the percentage of patients with AF that are routinely tested for SUA levels, how common hyperuricemia is in this population, and whether the diagnosis of hyperuricemia in patients with AF may have additional diagnostic significance.

## 2. Materials and Methods

### 2.1. Study Design and Participants

The Polish Atrial Fibrillation (POL-AF) Registry is a multicenter, prospective, observational study including patients with atrial fibrillation (AF) from 10 cardiology centers: seven of them academic, two regional hospitals, and one military hospital. The study was registered in ClinicalTrials.gov as NCT04419012. The data were gathered from January to December 2019 for 2 full chosen weeks each month. The aim of the record was to obtain data concerning clinical characteristics of patients with AF. Subsequently, patients with AF who were hospitalized in the centers for both urgent and planned reasons, over 18 years of age, and suffering from arrhythmia documented with electrocardiographic examination or medical documents, were added to the record. To gather a group that well represents Polish cardiological reality, no clear exclusion criteria were defined; however, patients admitted to hospital to have ablation due to AF were not included in the record.

**Figure 1:**
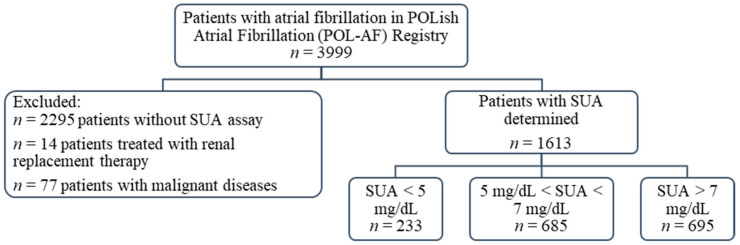
Flow chart of the study. Abbreviations: SUA, serum uric acid.

This study sub-analyzes the registry data on the importance of SUA levels in patients with AF. From the original group of 3999 patients included in the POL-AF registry, SUA concentration was determined in 1704 patients at the time of the index hospitalization. Patients treated with renal replacement therapy were excluded from this group (*n* = 14). Similarly, patients with malignant diseases—defined as people with active cancer or after oncology treatment completed less than a year before the date of registration—were excluded (*n* = 77). Ultimately, a group of 1613 patients was analyzed, including 705 women (44% of the studied population) (Figure 1).

Investigators collected baseline characteristics regarding demographics, medical history, type of AF, diagnostic test results, and pharmacotherapy. On the basis of creatinine concentration, the GFR value was calculated using MDRD rules. Cardiovascular risk was defined as high in patients with at least two of the following: hypertension, diabetes mellitus, previous stroke, atherosclerosis (including coronary atherosclerosis, myocardial infarction, peripheral arterial disease, coronary-aortic bypass graft, or atherosclerotic plaques), hypercholesterolemia, and chronic kidney disease (GFR < 60 mL/min/1.73 m^2^) [4,5]. The remaining patients were classified as NOT high cardiovascular risk. For the purposes of the analysis, two extreme values of uric acid concentration were adopted. The value of 7 mg/dL corresponds to the currently accepted upper limit of normal for this parameter in the general population [4,5]. However, they recommend that in the case of patients with cardiovascular diseases, the concentration of SUA should be below 5 mg/dL [4,5].

The study was approved by the Ethics Committee of the Swietokrzyska Medical Chamber in Kielce (104/2018). The Ethics Committee waived the requirement for informed consent from the patients.

### 2.2. Statistical Analysis

Data were described by means and standard deviations or frequencies and percentages when continuous or categorical, respectively. Due to the size of the studied population, for continuous variables a distribution similar to the normal distribution was assumed and parametric tests were applied. Group comparisons were performed using Student’s *t*-test for continuous variables and the chi-squared test for categorical variables. Pearson correlation was used to analyze the relationships between variables. Odds ratios (OR) with 95% confidence intervals (95% CI) were calculated in logistic regression models. In multivariate regression model, that included hypertension, diabetes, history of myocardial infarction, gender, SUA < 5 mg/dL, SUA > 7 mg/dL, and GFR < 60 mL/min/1.73 m^2^, multicollinearity between the covariates was checked. In order to determine the value of the cut-off points for the analyzed variables, the ROC method, Youden index, was used. Two-tailed *p*-values < 0.05 were considered statistically significant. All statistical analyses were performed using TIBCO Software Inc. Statistica 13 (Palo Alto, CA, USA).

## 3. Results

### 3.1. General Characteristics

The mean age of the subjects was 72 years (SD ± 11.6 years) and the mean SUA concentration in the study population was 6.88 mg/dL (SD ± 1.93). There was no significant difference in SUA concentration between women and men (6.86 ± 1.98 mg/dL and 6.90 ± 1.86, respectively, *p* = 0.65). Among the most common comorbidities, the prevalence of arterial hypertension (83% of the respondents) and heart failure (67% of the respondents, but HFrEF in only 19%) was particularly noteworthy, while coronary heart disease and chronic kidney disease were each present in half the patients. The general characteristics of the study population are presented in Table 1.

### 3.2. Uric Acid

In 43% of the subjects from the entire group, the concentration of SUA was >7 mg/dL, while values <5 mg/dL were found in only 14% of the subjects. Taking into account the current expert opinion on asymptomatic hyperuricemia, an additional division of the study population was made in terms of cardiovascular risk. This risk was assessed as high in 1362 patients (84%): in this group, SUA concentrations <5 mg/dL were found in only 14% of patients, while in 45% the concentration of SUA was >7 mg/dL. The group with NOT high cardiovascular risk included 251 patients (16%): 41% of these patients had SUA levels >7 mg/dL, and only 16% had levels <5 mg/dL.

Table 2 presents a comparison of two subgroups of the study population: patients with SUA concentration <5 mg/dL (223 patients, 14% of the entire study group) and patients with SUA concentration >7 mg/dL (695 patients, 45% of the entire study group). The mean concentrations of SUA in these subgroups were 4.37 and 8.59 mg/dL, respectively. New-onset AF occurred with similar frequency in both subgroups: 24 (10%) 57 (8%), respectively (*p* = 0.325).

In terms of the basic characteristics, patients with low concentrations of SUA had better renal function (mean creatinine concentration 1.14 vs. 1.28 mg/dL, *p* < 0.01; mean GFR values 69 mL/min/1.73 m^2^ vs. 58 mL/min/1.73 m^2^, *p* < 0.01), as well as lower concentrations of total cholesterol and triglycerides and a higher mean EF value (50 vs. 47%, *p* < 0.01). In terms of pharmacotherapy, aldosterone antagonists and other diuretics were used significantly more often in patients with SUA > 7 mg/dL. Moreover, significant differences were observed in the range of the ejection fraction between the groups—in the SUA > 7 mg/dL group there were significantly more patients with reduced EF, while in the SUA < 5 mg/dL group there were significantly more patients with preserved EF. This result prompted further analysis of the relationship between SUA concentration and EF.

### 3.3. Markers of Lowered Ejection Fraction of the Left Ventricle

Correlation analysis showed that SUA concentration was inversely correlated with left ventricular ejection fraction (LVEF). However, a separate analysis for the subgroups with SUA concentration <5 mg/dL and >7 mg/dL showed that the inverse correlation of SUA with the EF value was statistically significant only in patients with hyperuricemia (Table 3).

**Figure 2:**
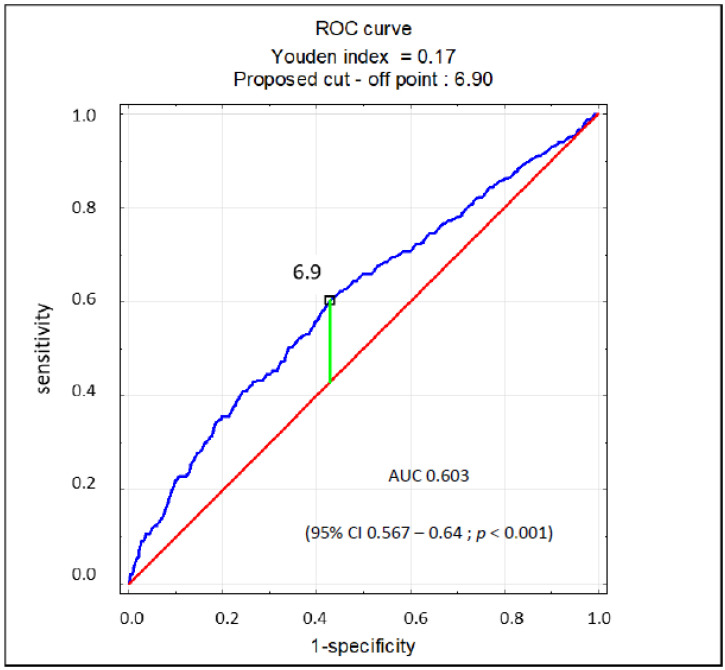
ROC curve for prognostic value of SUA concentration for EF < 40%.

The regression analysis revealed that SUA > 7 mg/dL in patients with AF is a significant marker of EF < 40% (odds ratio [OR] 1.89; 95% confidence interval [CI] 1.47–2.44; *p* < 0.001), while SUA < 5 mg/dL significantly indicates the opposite (OR 0.63; 95% CI 0.42–0.94; *p* = 0.02). SUA as a continuous variable also showed to be a significant marker for EF < 40% (OR 1.21; 95% CI 1.14–1.29; *p* < 0.001). In the multivariate analysis, UA > 7 mg/dL and GFR < 60 mL/min/1.73 m^2^ proved to be markers of EF < 40%. Female sex was a protective factor, but SUA < 5 mg/dL was not. The results of this analysis are presented in Table 4. There was no multicollinearity between the covariates in the regression model. Based on the ROC curve, the SUA concentration of 6.9 mg/dL was found to be the cut-off point for the reduced ejection fraction (EF < 40%) in patients with AF, characterized by 60% sensitivity and specificity (AUC 0.603; 95% CI 0.567–0.64; *p* < 0.001) (Figure 2).

## 4. Discussion

The main findings of this study are a high prevalence of hyperuricemia in patients with AF, and that hyperuricemia, specifically SUA > 6.9 mg/dL, may be a marker of reduced left ventricular EF in patients with AF. Tamariz et al. reported an increase in the risk of AF in patients with hyperuricemia. The analysis of data collected as part of the prospective ARIC cohort study showed that individuals with a SUA concentration >7 mg/dL have a two-fold higher risk of developing AF than those with a SUA concentration <5 mg/dL (HR 2.00; 95% CI 1.58–2.52) [16]. Zhang et al.’s meta-analysis of six cohort studies showed that hyperuricemia increases the risk of AF occurrence by nearly 50% (RR 1.49, 95% CI 1.24–1.79, *p* < 0.001) [17]. The results of Tamariz et al. and Zhang et al. are consistent in principle with the meta-analysis by Xu et al. [18]. The latest research results based on the analysis of 11 studies (over 500,000 patients in total and nearly 7000 cases of AF) showed that the relationship between SUA concentration and the risk of AF is linear, that the risk increases significantly even at SUA concentrations above 5 mg/dL, and that for every additional 1 mg/dL it increases by 21% (RR 1.21; 95% CI 1.12–1.32; I2 = 78%) [19].

Thus, the relationship between hyperuricemia and the risk of AF is established. Therefore, the results of our own study indicating the frequent occurrence of hyperuricemia among patients included in the registry should come as no surprise. To the best of the authors’ knowledge, however, the importance of hyperuricemia in the case of an already diagnosed arrhythmia has not yet been the subject of major research. The data collected as part of the POL-AF registry make it impossible to prospectively assess the role of hyperuricemia in the prognosis of patients with atrial fibrillation. The study protocol did not include follow-up, and the number of in-hospital deaths was too small for reliable statistical analyses. However, the observation of a significant relationship between hyperuricemia and lowered ejection fraction in patients with AF prompts the analysis of data on the association of SUA with heart failure. 

This relation between SUA and HF is known, although widely debated and ambiguous. Huang et al., in a meta-analysis of 28 studies on HF and hyperuricemia, have shown that every 1 mg/dL increase in SUA concentration gives a 19% (HR 1.19, 95% CI 1.17–1.21) increase in the risk of HF occurrence [20]. In addition, an increase in UA concentration by 1 mg/dL translates into an increase in risk of all-cause mortality of 4% (HR 1.04, 95% CI 1.02–1.06) and in the risk of composite endpoint in HF patients by 28% (HR 1.28, 95% CI 0.97–1.70) [20]. In a recent study, Huang and colleagues have also shown that in patients with HF, hyperuricemia increases the risk of all-cause mortality by 43% (RR 1.43; 95% CI 1.31–1.56) and the risk of combined endpoint of death or readmission by 68% (RR 1.68; 95% CI 1.33–2.13). They also found that each 1 mg/dL increase in SUA significantly increased, by 11 and 12%, the risk of all-cause mortality and the combined endpoint of death or readmission, respectively [21].

On the other hand, a recently published meta-analysis by Kanbey et al. showed that the use of allopurinol in a patient with HF worsens the prognosis (increase in all-cause mortality, 24%; in cardiovascular mortality, 42%) [22]. However, heart failure is a disease of many paradoxes—we know that in this population there is no longer any benefit in reducing LDL levels, and the obesity paradox is well known [23,24].

Discrepancies in the results of studies on the prognostic significance of hyperuricemia in heart failure and the effects of uricosuric drugs indicate the need to analyze the pathophysiological mechanisms explaining the relationships between hyperuricemia and AF and hyperuricemia and HF. The main questions to be asked here are (1) when is hyperuricemia a risk factor for the occurrence of myocardial damage, and (2) when is it a marker of damage that has already occurred. One of the hypothetical postulated mechanisms is an increase in the activity of the local renin–angiotensin–aldosterone system under the influence of high SUA concentrations [25]. In the animal model, an increase in the concentration of renin mRNA was also observed, due to the high concentration of SUA [25,26]. Another hypothesis is that cardiac remodeling, which increases the risk of arrhythmias, is associated with excessive xanthine oxidase (XO) activity and oxidative stress [15]. Blocking XO with allopurinol has been shown in an animal model to reduce the risk of AF occurrence [14]. There is also evidence of the cardiotoxicity of uric acid accumulated by cells, which may lead not only to structural remodeling of the heart but also to proarrhythmic imbalance between ion channels (electrical remodeling) [14,15,25]. The results of studies proving the antagonistic effect of uric acid against nitric oxide and NO synthase are also very interesting and disturbing; this may be the mechanism of SUA toxicity toward endothelial cells [14,15,25].

When analyzing the occurrence of hyperuricemia, attention must be paid to the impaired renal function. In our study, half the patients had an eGFR < 60 mL/min/m^2^. Patients in the group with SUA > 7 mg/dL had significantly worse renal parameters, but extreme cases (patients on dialysis and patients with active oncological disease) were excluded from the analysis. Decreased eGFR is a marker of reduced EF. However, bearing in mind the correlation of heart failure and renal failure, it is difficult to judge whether high SUA is due more to HF or to CKD. 

Kumric, in a recent review concerning HF and SUA, has identified two main mechanisms of hyperuricemia in this patient population: the hypoxia typical of progressive HF, catabolism, and cell apoptosis, and frequent insulin resistance stimulate XO activities. Excessive XO activity stimulates oxidative stress, which in turn is also stimulated by tissue hypoperfusion. As a result, there is simultaneous overproduction of UA and a complex cardiotoxic effect [27]. On the other hand, excretion of UA in the urine is impaired. This is partly a consequence of an increased concentration of lactic acid (secondary to tissue hypoxia), partly caused by commonly used HF drugs such as renin–angiotensin–aldosterone inhibitors and diuretics, and also due to the effect of ischemia of the kidneys themselves (cardio-renal syndrome) [27].

The observations of some authors suggest that in the case of already diagnosed HF, SUA may be a marker of its advancement [14]. This is proven by the positive correlations between SUA and the severity of clinical symptoms of HF. The results of our analysis, showing a clear relationship between SUA concentration and reduced ejection fraction in patients with AF, are also a part of this thinking. Our results indicate the validity of the determination of SUA concentration in patients with AF as a potential marker of reduced ejection fraction and, therefore, also as a potential negative prognostic marker.

## 5. Conclusions

Nearly half (43%) of patients with AF have elevated SUA levels (UA > 7 mg/dL). Among patients with AF at high cardiovascular risk, few have UA concentration <5 mg/dL, and in nearly half (45%) the UA concentration is >7 mg/dL. Hyperuricemia seems to be a frequent phenomenon, yet this parameter is determined in less than every second patient with AF. It seems that elevated SUA levels may indicate the presence of reduced left ventricular ejection fraction in patients with AF. The cut-off value (6.9 mg/dL) is close to the UA concentration currently considered pathological. In view of the contradictory data on the impact of uric acid-lowering therapy on the prognosis of patients with HF, the results of our analysis are primarily of cognitive value. However, from a practical point of view, in outpatient care or in the case of a diagnosis of AF in non-cardiology departments, the diagnosis of hyperuricemia may be a prerequisite for an accelerated ECHO test. Hence, further studies on the relationship between hyperuricemia and the impairment of the left ventricular ejection fraction in patients with atrial fibrillation seem justified.

## 6. Limitations

Our study has some limitations. First, the registry protocol does not provide for prospective follow-up. This fact and the very low number of in-hospital deaths made it impossible to assess the relationship between SUA and the prognosis of patients with AF. The relationship between hyperuricemia and the applied regimen has not been analyzed; in particular, no information on the use of drugs that reduce SUA was available. We also did not take into account the potential impact of other drugs that may increase SUA levels (e.g., diuretics, ACE inhibitors, etc.). The registry did not analyze the doses of these drugs or collect information on the specific molecules used. Moreover, SUA levels were missing in 57% of the registry patients. This may have introduced selection bias into the analysis, as the analysis may have excluded the healthier AF patients (that is, those for whom the determination of SUA was not deemed necessary). However, the authors are of the opinion that this is rather the effect of differences in standard sets of laboratory tests between individual centers.

Despite these limitations, to our knowledge, this is the first such large study to assess the incidence of hyperuricemia in patients with AF and to indicate the potential role of SUA as a marker of reduced EF in these patients.

## Figures and Tables

**Table 1:** General characteristics of study group (*n* = 1613).

**Demographics**		**Number (%) or Mean (±SD)**	
Women		705 (44)	
Age (years)		71.98 (±11.61)	
BMI (kg/m^2^)		29.35 (±5.60)	
SUA < 5 mg/dL		233 (14)	
SUA > 7 mg/dL		695 (43)	
High cardiovascular risk *		1362 (84)	
**Comorbidities**		**Number (%)**	
Arterial hypertension		1343 (83)	
Heart failure		1087 (67)	
HFrEF (EF < 40%)		300 (19)	
Diabetes		539 (33)	
History of stroke		190 (12)	
History of TIA		95 (6)	
CAD		806 (50)	
Post CABG		121 (7.5)	
Post PCI		365 (23)	
History of MI		355 (22)	
PAD		232 (14)	
CKD (MDRD < 60)		812 (50)	
**Laboratory Results (unit)**	**Mean**		**Standard Deviation**
HGB (g/dL)	13.25		1.88
GFR (mL/min/1.73 m^2^)	62.24		22.12
ALT (U/L)	31.05		52.03
AST (U/L)	33.48		50.10
SUA (mg/dL)	6.88		1.93
TC (mg/dL)	167.47		52.40
LDL (mg/dL)	97.93		43.67
HDL (mg/dL)	47.60		22.12
TG (mg/dL)	124.88		61.75
EF (%)	48.84		13.34

Abbreviations: BMI, body mass index; HGB, hemoglobin; GFR, glomerular filtration rate; ALT, alanine aminotransferase; AST, aspartate aminotransferase; SUA, serum uric acid; TC, total cholesterol; LDL, low-density lipoproteins; HDL, high-density lipoproteins; TG, triglycerides; EF, ejection fraction; HFrEF, heart failure with reduced ejection fraction; TIA, transient ischemic attack; CAD, coronary arterial disease; CABG, coronary-aortic bypass graft; PCI, percutaneous coronary intervention; MI, myocardial infarction; PAD, peripheral arterial disease; CKD, chronic kidney disease. * High cardiovascular risk defined as in Borghi et al. and described in the Materials and Methods section [4,5].

**Table 2:** Comparison of basic characteristics of subgroups with low (SUA < 5 mg/dL) and high (SUA > 7 mg/dL) uric acid concentrations.

Parameter (Unit)	SUA > 7 (*n* = 695)		SUA < 5 (*n* = 233)		*p* Value
	Mean	SD	Mean	SD	
Age (years)	71.94	11.85	72.23	11.05	0.733
BMI (kg/m^2^)	28.90	5.54	29.73	5.36	0.078
Creatinine (mg/dL)	1.28	0.43	1.14	0.62	<0.001
GFR (mL/min/1.73 m^2^)	57.88	20.33	69.11	24.20	<0.001
UA (mg/dL)	8.59	1.45	4.37	1.04	<0.001
TC (mg/dL)	165.61	54.37	154.79	47.75	0.008
LDL (mg/dL)	97.01	45.28	90.59	40.73	0.060
HDL (mg/dL)	46.01	24.04	46.78	13.45	0.649
TG (mg/dL)	129.75	66.21	100.88	46.81	<0.001
EF (%)	46.83	14.47	50.17	11.49	0.003
**EF ranges**	***n* (%)**		***n* (%)**		***p* value**
EF < 40%	168 (24)		31 (13)		<0.001
EF ≥ 50%	325 (47)		132 (57)		0.001
EF 40–50%	91 (13)		29 (12)		0.874
No ECHO	111 (16)		41 (18)		0.561
**Medications**	***n* (%)**		***n* (%)**		***p* value**
Beta-blockers	198 (85)		609 (88)		0.384
ACE-I	134 (57,5)		417 (60)		0.608
Sartans	41 (18)		151 (22)		0.190
Aldosteron antagonists	79 (34)		325 (47)		<0.001
Other diuretics	135 (56)		518 (74,5)		<0.001
Digoxin	24 (10)		68 (10)		0.790

Abbreviations: BMI, body mass index; GFR, glomerular filtration rate; SUA, serum uric acid; TC, total cholesterol; LDL, low-density lipoproteins; HDL, high-density lipoproteins; TG, triglycerides; EF, ejection fraction; SD, standard deviation; ACE-I, angiotensin convertase inhibitors. Significant difference are marked in red.

**Table 3:** (**A**–**C**). Results of correlation analysis of the whole group (**A**) and of subgroups with low SUA (**B**) and high SUA (**C**) concentration.

**A Correlations for the Whole Study Group**								
**Correlation index (r)**	**SUA**	**Age**	**Cr**	**GFR**	**LDL**	**HDL**	**TG**	**EF**
SUA (mg/dL)	1.00	−0.03	0.23	−0.24	−0.04	−0.10	0.06	−0.19
Age (years)	−0.03	1.00	−0.04	−0.14	−0.04	0.00	0.03	−0.01
Cr (mg/dL)	0.23	−0.04	1.00	−0.75	−0.06	−0.09	0.02	−0.20
GFR (mL/min/1.73 m^2^)	−0.24	−0.14	−0.75	1.00	0.03	0.05	−0.05	0.17
LDL (mg/dL)	−0.04	−0.04	−0.06	0.03	1.00	0.08	0.38	0.10
HDL (mg/dL)	−0.10	0.00	−0.09	0.05	0.08	1.00	0.00	0.11
TG (mg/dL)	0.06	0.03	0.02	−0.05	0.38	0.00	1.00	0.08
EF (%)	−0.19	−0.01	−0.20	0.17	0.10	0.11	0.08	1.00
**B Correlations for UA < 5**								
**Correlation index (r)**	**SUA**	**Age**	**Cr**	**GFR**	**LDL**	**HDL**	**TG**	**EF**
SUA (mg/dL)	1.00	0.00	0.09	−0.04	−0.05	−0.17	−0.03	−0.01
Age (years)	0.00	1.00	−0.02	−0.06	−0.08	0.13	0.00	0.13
Cr (mg/dL)	0.09	−0.02	1.00	−0.08	−0.06	−0.11	0.15	−0.26
GFR (mL/min/1.73 m^2^)	−0.04	−0.06	−0.08	1.00	0.01	0.08	−0.09	0.04
LDL (mg/dL)	−0.05	−0.08	−0.06	0.01	1.00	0.14	0.29	−0.02
HDL (mg/dL)	−0.17	0.13	−0.11	0.08	0.14	1.00	−0.14	0.12
TG (mg/dL)	−0.03	0.00	0.15	−0.09	0.29	−0.14	1.00	−0.10
EF (%)	−0.01	0.13	−0.26	0.04	−0.02	0.12	−0.10	1.00
**C Correlations for UA > 7**								
**Correlation index (r)**	**SUA**	**Age**	**Cr**	**GFR**	**LDL**	**HDL**	**TG**	**EF**
SUA (mg/dl)	1.00	−0.03	0.20	−0.12	−0.14	−0.15	−0.06	−0.18
Age (years)	−0.03	1.00	−0.03	−0.17	−0.06	0.00	0.01	−0.03
Cr (mg/dL)	0.20	−0.03	1.00	−0.78	−0.16	−0.07	0.00	−0.20
GFR (mL/min/1.73 m^2^)	−0.12	−0.17	−0.78	1.00	0.15	0.02	−0.01	0.17
LDL (mg/dL)	−0.14	−0.06	−0.16	0.15	1.00	0.10	0.39	0.15
HDL (mg/dL)	−0.15	0.00	−0.07	0.02	0.10	1.00	0.01	0.11
TG (mg/dL)	−0.06	0.01	0.00	−0.01	0.39	0.01	1.00	0.15
EF (%)	−0.18	−0.03	−0.20	0.17	0.15	0.11	0.15	1.00

GFR, glomerular filtration rate; SUA, serum uric acid; TC, total cholesterol; LDL, low-density lipoproteins; HDL, high-density lipoproteins; TG, triglycerides; EF, ejection fraction; Cr, creatinine; r, correlation index (Pearson’s). Significant positive and negative correlations are marked in green and red, respectively (*p* < 0.05).

**Table 4:** Markers of EF < 40%. Results of multivariate regression analysis.

	OR	95% CI	*p* Value
SUA < 5 mg/dL	0.966	0.624–1.496	0.877
SUA > 7 mg/dL	1.741	1.319–2.298	<0.001
GFR < 60 mL/min/1.73 m^2^	1.939	1.459–2.578	<0.001
AH	1.155	0.812–1.641	0.423
DM	0.952	0.720–1.260	0.731
History of MI	1.071	0.780–1.471	0.671
Female sex	0.735	0.556–0.971	0.030

SUA, serum uric acid; GFR, glomerular filtration rate; AH, arterial hypertension; DM, diabetes; MI, myocardial infarction. Significant difference are marked in red.

## Data Availability

Data are contained within the article.
